# The synergistic effect of concussions and aging in women? Disparities and perspectives on moving forward

**DOI:** 10.2217/cnc-2018-0004

**Published:** 2018-08-15

**Authors:** Carrie Esopenko, Adrienne H Simonds, Ellen Z Anderson

**Affiliations:** 1 Department of Rehabilitation & Movement Sciences, School of Health Professions, Rutgers, The State University of New Jersey, New Brunswick, NJ, 08901, USA; 2 Department of Health Informatics, School of Health Professions, Rutgers, The State University of New Jersey, New Brunswick, NJ, 08901, USA

**Keywords:** aging, chronic traumatic encephalopathy, concussion, dementia, women

Sports-related concussion is a significant health concern affecting approximately 1.6–3.8 million people in the USA each year [1]. Although male athletes are exposed to more contact in sports [2], rates of concussion are higher for female athletes in sex-comparable sports at both the high school and collegiate level [2–4]. Furthermore, female athletes tend to report a greater number of symptoms and take longer to recover from concussion compared with male athletes [4–6]. Impaired cognitive function and alterations in brain structure and function have been noted in the acute phase after injury in both males and females, and it has been reviewed in depth recently [5,7,8]. However, the majority of research assessing the long-term implications of mild traumatic brain injury (TBI) or sports-related concussion has been limited to male athletes.

Past studies have suggested that a history of TBI increases the risk of a number of neurodegenerative diseases, including Alzheimer's disease [9,10], Parkinson's disease [11], amyotrophic lateral sclerosis [12,13], frontotemporal dementia [14] and chronic traumatic encephalopathy (CTE) [15–17]. With the discovery of CTE in male athletes with a history of repetitive brain trauma [15–17], considerable focus has been placed on the link between sports-related concussions and the development of CTE.

CTE, originally termed dementia pugilistica, was first discovered by Harrison Martland in boxers, whom he described as being punch drunk [18]. A further series of cases described by Corsellis and colleagues noted a similar pattern of histological and cerebral changes in boxers [19]. In 2005, Dr. Bennet Omalu discovered neuropathological changes characteristic of CTE in a retired professional football player, who died due to myocardial infarction [15]. A recent postmortem study found CTE in 177 of the 202 brains examined, all of whom were deceased subjects who participated in contact sport with repetitive exposure to brain trauma [20]. Furthermore, a retrospective autopsy series of individuals whose brains were donated to a neuropathology brain bank found that a third of individuals with a history of participation in contact sports showed evidence of CTE; no autopsies on individuals without a history of contact sports showed evidence of CTE [21]. It should be noted that recent work has also found CTE pathology in those without a history of repetitive brain trauma [22,23]

CTE is characterized by the accumulation of hyperphosphorylated tau in the depths of sulci, that progresses from the frontal to temporal cortical regions [17]. A thorough discussion of CTE pathology can be found in the following references [16,17,20,24]. Although CTE cannot yet be diagnosed in the living, retrospective medical and mental health histories from family members of those diagnosed with CTE suggests that there is a characteristic cognitive and behavioral profile of impairments dependent upon when disease progression begins [25]. However, to date, no female athlete has been diagnosed with CTE, and furthermore, no prospective *in vivo* or neuropathological studies have assessed the overall long-term effects of concussions in female athletes. Thus, we cannot begin to assess whether women are at risk of developing CTE. Given the prevalence of concussion in female athletes, it would be expected that female athletes are just as susceptible to multiple or repetitive concussions as male athletes. Therefore, unless women possess a universal protective factor that decreases their risk of developing CTE, one can assume that female athletes are also at risk.

In addition to females having higher rates of sports-related concussions, women also have higher rates of neurodegenerative disease, particularly Alzheimer's disease, relative to men [26]. A recent meta-analysis found that 35.6 million people are living with dementia worldwide, with a higher prevalence of dementia found in women [27]. With the number of individuals living with dementia projected to increase to 115 million by 2050 [27], there will be a significant indirect and direct burden of dementia on society. Although TBI has been identified as one risk factor affecting the development and age of the onset of dementia [28], no research has examined the interaction between sports-related concussion and the development of neurodegenerative disease in women. However, a recent cohort study found that the history of TBI was associated with increased odds of developing dementia which was higher in those with repetitive TBI; the odds of developing dementia after TBI was similar in males and females [29]. As such, one can expect a similar relationship occurring for males and females with a history of sports-related concussion, particularly when repetitive injuries have occurred, again highlighting the importance of examining the long-term effects of these injuries in women.

Past research has also examined the relationship between concussions and later-life psychiatric, cognitive, and neurological impairments, with studies to date showing mixed results. However, one significant limitation of this research is that it has all been done in male athletes. Nonetheless, these studies suggest that remote concussion is associated with an increased risk of mild cognitive impairment [30], depression [31], and psychiatric symptoms [32] during aging. Similarly, a history of subconcussive impacts has been associated with later-life cognitive impairments in retired American professional football players [33]. In addition, earlier life exposure to head impacts may also be associated with greater later-life cognitive impairment and altered white matter integrity [34,35]. Yet, other work has suggested a limited relationship between the history of repetitive concussions and cognitive impairments in later-life in former international rugby [36] and professional hockey players [32], or found no significant association between playing high school football and cognitive impairment and depression during aging [37].

Concussions also seem to have subsequent downstream consequences on brain function and structure during aging; yet again, these studies are similarly limited in that all have been done in male athletes. Still, these studies have shown alterations in brain function [38–40], evidence of volumetric changes such as cortical thinning [41,42], and loss of white matter integrity [42,43] in male athletes years after concussion. Alterations in brain function and structure have also been shown in former American high school and college football players with cognitive deficits and depression, although these changes were not related to concussion history [44]. However, recent work from Terry and colleagues did not find a relationship between mild TBIs in high school football players and brain atrophy in later life [45].

Together, the literature suggests a potential synergistic relationship between repetitive brain trauma and later-life brain and behavioral outcomes in male athletes who participated in collegiate or professional sports; however, our knowledge of the long-term consequences of sports-related concussion on brain and behavioral function in female athletes is severely lacking. As it stands, we are ignoring a significant proportion of the population in studies conducted thus far assessing the interaction between repetitive brain trauma and aging. This topic can no longer be ignored, particularly given the known sex-related physiological differences that occur during aging, as well as reports that females have longer standing post-concussion symptoms compared to males [4,6–8,46,47].

Considering the higher rates of sports-related concussion in females competing in sex-comparable sports, coupled with women having an increased risk of developing dementia, we advocate for the development of prospective studies specifically targeting women to assess the interaction between concussion, repetitive exposure to brain trauma, and the potential brain and behavioral changes that occur across the lifespan. Such studies should include comprehensive neuropsychological and neurological evaluations, which also consider genetic risk factors, tracking of menstrual cycle function post-injury, and the age of injury, to better inform the long-term implications of these injuries, as well as their impact on sex-specific health outcomes, such as fertility and/or childbearing.

Based on past research, we highlight a few specific factors that should be incorporated in prospective studies aimed at understanding the mechanisms underlying sex differences in sports-related concussion outcomes, as well as their long-term effects. The first factor is the role specific genetic polymorphisms play in this relationship, given that genetic polymorphisms have been linked to concussion recovery and long-term outcomes, with most work focusing on *ApoE*. In particular, presence of the *ApoE ε4* allele has been linked to poorer outcomes following concussion and TBI [48]. In college athletes, the presence of the *ApoE ε4* allele was also associated with impaired neurocognitive function in concussed athletes [49], as well as a greater number of symptoms post-concussion [50]. Other studies, however, have failed to find an association between *ApoE ε4* and outcomes following concussion and TBI [51–53]. Recent work has also shown that genetic polymorphisms other than the *ApoE ε4* allele may be linked to concussion outcomes. For example, there is evidence that *BDNF*, *ApoE promotor –219 G/T* and *GRIN2A* gene promoter may be associated with poorer prognosis following concussion [53–56]. However, the influence specific genetic polymorphisms have on concussion outcomes and their long-term effects, dependent on sex, have not been examined.

In addition, specific genotypes, such as the presence of the *ApoE ε4* allele, may also increase the risk of developing dementia in individuals with remote history of TBI [57–59]. *ApoE ε4* on its own has been shown to increase the risk of developing dementia twofold, with the risk increasing to tenfold in individuals who possess the *ApoE ε4* allele with a history of TBI [57]. However, in the seminal work from McKee and colleagues, the proportion of athletes with CTE with at least one *ApoE ε4* allele did not differ from the general population, although this association was examined in a small number of samples [17]. Furthermore, Esopenko and colleagues found that having at least one copy of the Apo*E ε4* allele was associated with increased psychiatric complaints in a small sample of retired professional male hockey players [32]. Although there seems to be a stronger relationship between the *ApoE ε4* allele and the development of Alzheimer's disease in women [60,61], the interaction between sports-related concussion, genetic polymorphisms, and sex on the development of dementia has not been explored.

The influence of gonadal sex hormones is another factor that we believe to be of significance, both in understanding acute and chronic concussion outcomes, but also in the long-term effects of these injuries. Although limited, past research suggests that sex hormones may influence symptom prevalence and severity in females post-concussion, with anterior pituitary deficiencies [62], disruption of the hypothalamic-pituitary-ovarian axis [63], and progesterone withdrawal [64] being proposed as potential mechanisms. A recent study by Snook and colleagues identified abnormal menstrual patterns in young women after concussion, which they propose is due to disruption of the hypothalamic-pituitary-ovarian axis [63]. Furthermore, others have shown poorer outcomes when TBI occurred during the follicular phase of the menstrual cycle, an environment of high progesterone levels, compared with the luteal phase, when progesterone levels are low [64]. Wunderle and colleagues suggest that poorer outcomes are the result of progesterone withdrawal due to the large decrease in progesterone when injury occurs in the follicular phase compared with the luteal phase. Furthermore, past studies have shown that estrogen and progesterone affect outcomes following moderate-to-severe TBI, with the age of injury affecting the influence of these hormones on recovery [65,66]. Other studies postulate that progesterone is protective in TBI and it has been suggested as a potential treatment following TBI [67,68]. Moreover, gonadal sex hormones are thought to play a role in the development of dementia, either through protection or enhanced risk [69,70]. As such, future research should assess the influence of sex hormones on acute concussion outcomes, as well as consider the role of these hormones on the long-term outcomes of concussion during aging. These studies will also need to account for the age of injury, given changes in sex hormones across the lifespan (i.e., menopause).

Finally, there has been a strong push to have current and former athletes donate their brains for future research through the development of brain banks. Brain banks examine neuropathological changes in the brain postmortem in those who have either donated their brains during life, or have had their brains donated by family members upon their death. Although brain banks provide incredibly important insights into the neuropathology of disease, they tend to lack prospective measures of factors underlying the development of disease and disease progression (e.g., substance use, undiagnosed mental illness). So far, most neuropathological studies have attempted to account for these factors through responses from family members of the deceased; however, these responses are inherently biased, particularly from family members who have donated their loved ones’ brains due to fear that they had CTE or dementia. Nonetheless, brain banks have been extremely important in understanding CTE, developing stages of disease progression, and identifying potential clinical and behavioral complaints in those with CTE, but again, the outcomes from these studies have all been limited to male athletes. We are, however, making positive strides in this area, as recently it was announced that a female brain bank would be initiated through the National Posttraumatic Stress Disorder Brain Bank to examine the relationship between head injury and dementia, specifically in women. Further, a number of current and former female athletes have agreed to donate their brains for autopsy studies. As in men, this will yield important insights into the effect concussions and TBI have during aging, and, importantly, whether CTE pathology occurs in women. While these developments represent a movement in the right direction, we should also be ensuring that males and females who agree to donate their brains participate in longitudinal studies to collect information about *in vivo* factors that influence the development and progression of disease.

Taken together, although our understanding of how concussions affect the aging process is still in its infancy, there is limited evidence to suggest a synergistic effect between concussions and aging on risk of neurodegenerative disease, as well as alterations in brain and behavioral function in later-life. Yet, as it stands, a significant proportion of the population who have exposure to concussion, and who, based on past research, may be at a heightened risk of experiencing long-term impairments from these injuries, have been ignored. As such, it is imperative that future research employs prospective studies to not only determine whether concussions are associated with different brain and behavioral outcomes in male and female athletes, but also how concussions affect women longitudinally.
